# Unbalanced sex-ratio in the Neolithic individuals from the Escoural Cave (Montemor-o-Novo, Portugal) revealed by peptide analysis

**DOI:** 10.1038/s41598-023-47037-4

**Published:** 2023-11-14

**Authors:** Raquel Granja, Ana Cristina Araújo, Federico Lugli, Sara Silvestrini, Ana Maria Silva, David Gonçalves

**Affiliations:** 1 Laboratory of Archaeosciences (LARC/CIBIO/InBIO), Direção-Geral do Património Cultural, Calçada do Mirante à Ajuda n.º 10, 1300-418 Lisbon, Portugal; 2 Research Centre for Anthropology and Health (CIAS), Department of Life Sciences, University of Coimbra, Calçada Martim de Freitas, 3000-456 Coimbra, Portugal; 3 Centre for Archaeology, University of Lisbon (UNIARQ), Faculty of Humanities, University of Lisbon, Alameda da Universidade, 1600-214 Lisbon, Portugal; 4 Department of Chemical and Geological Sciences, University of Modena and Reggio Emilia, Modena, Italy; 5 Institut für Geowissenschaften, Goethe-Universität Frankfurt, Frankfurt Am Main, Germany; 6 BONES Lab, Department of Cultural Heritage, University of Bologna, Via Degli Ariani 1, 48121 Ravenna, Italy; 7 Laboratory of Forensic Anthropology, Centre for Functional Ecology-Science for People & the Planet (CFE), Department of Life Sciences, University of Coimbra, Calçada Martim de Freitas, 3000-456 Coimbra, Portugal

**Keywords:** Biological techniques, Molecular biology, Proteomics

## Abstract

The sex profile estimation of pre-historic communities is often complicated by the commingled and scattered nature of skeletal assemblages. Demographic profiles are usually lacking and provide very truncated representations of these populations but proteomic analysis of sex-specific amelogenin peptides in tooth enamel brings new promise to these studies. The main objective was to obtain the sex profile of the human assemblage recovered from the Neolithic cave-necropolis of Escoural (Montemor-o-Novo, southern Portugal) through liquid chromatography-mass spectrometry. The secondary objective was to analyse sex-specific linear enamel hypoplasias (LEH), and to test the reliability of canine odontometric sex estimation. Sex estimation through peptide analysis was carried out in 36 left permanent canines which were macroscopically examined for the presence of LEH. The canine buccolingual diameter was used for odontometric sex estimation. The obtained sex ratio (0.5:1, M:F) is biased to female individuals, probably due to cultural factors since the natural sex ratio of the human population falls between 0.95:1 and 1.02:1 (M:F). A high frequency of LEH was observed, but with no significant sexual differences (*p* = 0.554). The mean LEH age of onset occurred at 3 years of age, with no significant differences between the sexes (*p* = 0.116), and was possibly related to the weaning process. Odontometric sex estimation revealed a correct classification of 80%, with a high number of males mistakenly attributed to females. This study is one of the largest samples subjected to peptide analysis, and thus demonstrates its usefulness on the research of commingled and scattered skeletal assemblages.

## Introduction

Biological profiling of individuals from prehistoric necropolises often poses challenges to biological anthropologists due to the usual commingled, fragmented, and scattered disposition of human skeletal remains^1–8^. In such cases, sex estimation is relatively challenging because it must combine sexually dimorphic skeletal element that simultaneously provides the highest possible minimum number of individuals (MNI). This is a common scenario in prehistoric funerary contexts due to complex social, environmental, and biological taphonomic factors^9^ and is also the case of the Neolithic Escoural Cave with a MNI of 109 (42 immatures and 67 matures) located in the Alentejo (Montemor-o-Novo, southern Portugal), for which the sex profile is discussed in this paper.

In both biological and forensic anthropology, sex estimation is a key element in the investigation of the biological profile, along with age at death, stature, and ancestry^10,11^. In turn, age at death, stature and ancestry estimates are sex-dependant^12–14^, which makes sex assessment even more relevant. Its importance relies also in the fact that it is critical for palaeodemographic reconstructions and provides better insight about the activities of human past populations, namely funerary practices^3–7,15–20^.

Ancient DNA still does not constitute an easy and widespread solution to the sex profiling of collective necropolises since it requires a high sample consumption which does not contribute to the preservation of archaeological materials, and is highly sensitive to the DNA contamination during the excavation, material manipulation, and analysis, besides being too expensive, time-consuming, and requiring good preservation of the material^21,22^. Therefore, conventional skeletal sex estimation is still the most accessible approach to study the sex profile of ancient populations. Metric and morphognostic skeletal sex estimations are based on sexual dimorphism in size and shape at the intra- and inter-population levels^23^ due to genetic and hormonal sexual differences starting early during foetal development at 7–9 gestational weeks^11,24,25^.

However, the sex estimation reliability of immature remains is much lower than that of adult remains and is therefore often under-studied leading to truncated sex profiles, i.e. excluding non-adult. The development of more reliable sex estimation methods applied to immature individuals is hampered by the usual reduced size of available reference samples^25^. Nevertheless, both metric and morphognostic^26–30^ methods have been proposed to estimate the sex of non-adults, with varied accuracies and a tendency towards better classification of males to the detriment of females (see review in^25^).

Metric methods have a serious drawback since they are population-specific and applying them to other populations can be problematic^31–33^. Another issue is that the most dimorphic portions of long bones, the epiphyses^34^, are often poorly preserved in archaeological contexts thus limiting their use. Immature remains are also known to present worse preservation. In turn, morphognostic methods are based on qualitative assessments of sexual indicators and/or their expressivity thus being more susceptible to intra and interobserver errors^32,35–39^. Sexual dimorphism is population-specific both at the temporal and spatial scales^40–42^ besides being also variable at the intra-population level^43^. Although the hip bone, which is the most sexually dimorphic bone, is not as much affected by these hindrances^41^, it is often not sufficiently preserved in collective tombs with commingled, fragmented, and scattered remains to allow for the widespan estimation of sex.

Preservation problems are less of an issue with teeth, which tend to preserve better than bones^2,5,7,44–47^. Pilloud and Scott^48^ showed that, among different populations, the most dimorphic measurement was the buccolingual diameter of the lower canine along with, to a smaller degree, the same measurement on the upper canine. In non-adults, these were also demonstrated to be the most sexually dimorphic features^27^. Therefore, and despite the limitations mentioned above, odontometry appears to be one potential option to achieve sex estimation of commingled, scattered, and poorly preserved remains in large assemblages, although only rarely this claim has been put to test in archaeological remains since no DNA or peptides confirmation are usually carried out in large scale^49^.

The ground-breaking potential of sex estimation through tooth enamel peptides has recently been demonstrated^50–58^ and encompasses several advantages. It is reliable, fast, inexpensive, and minimally destructive^50,58,59^. As mentioned above, tooth enamel is very resistant to taphonomic and diagenetic factors owing to its larger bioapatite crystals and higher mineral content compared to bone. Teeth are indeed among the skeletal elements providing highest MNIs owing to their better preservation^5,7,60–62^.

The advantages of teeth are not limited to sex estimation. Teeth record infancy’s non-specific physiological stress events through linear enamel hypoplasias (LEH hereafter) often caused by severe illness and/or nutritional deficiencies^63^. Since tooth crowns do not suffer remodelling, LEH are a good indicator of those events^64^ occurring during amelogenesis between the 6th gestational month and 18.5 years^65^ even though LEH age estimation is only roughly achieved, since dental wear is difficult to assess and measure^64,66,67^.

The main objective of this paper was to obtain the most complete as possible sex profile of the collection of human remains from the Neolithic cave-necropolis of Escoural, based on amelogenin analyses. Secondary objectives were to assess potential sex differences regarding LEH-monitored physiological stress events, and to test the reliability of canine odontometric sex estimation by comparing it with the sex classifications obtained through the peptides analysis. As a result, this paper provides a comprehensive portrait of the sex profile of the Escoural Cave-necropolis, which is inclusive of both adult and non-adult individuals, a feat never before achieved for Neolithic human populations inhabiting this Portuguese inner Alentejo region.

### Site description

**Figure 1:**
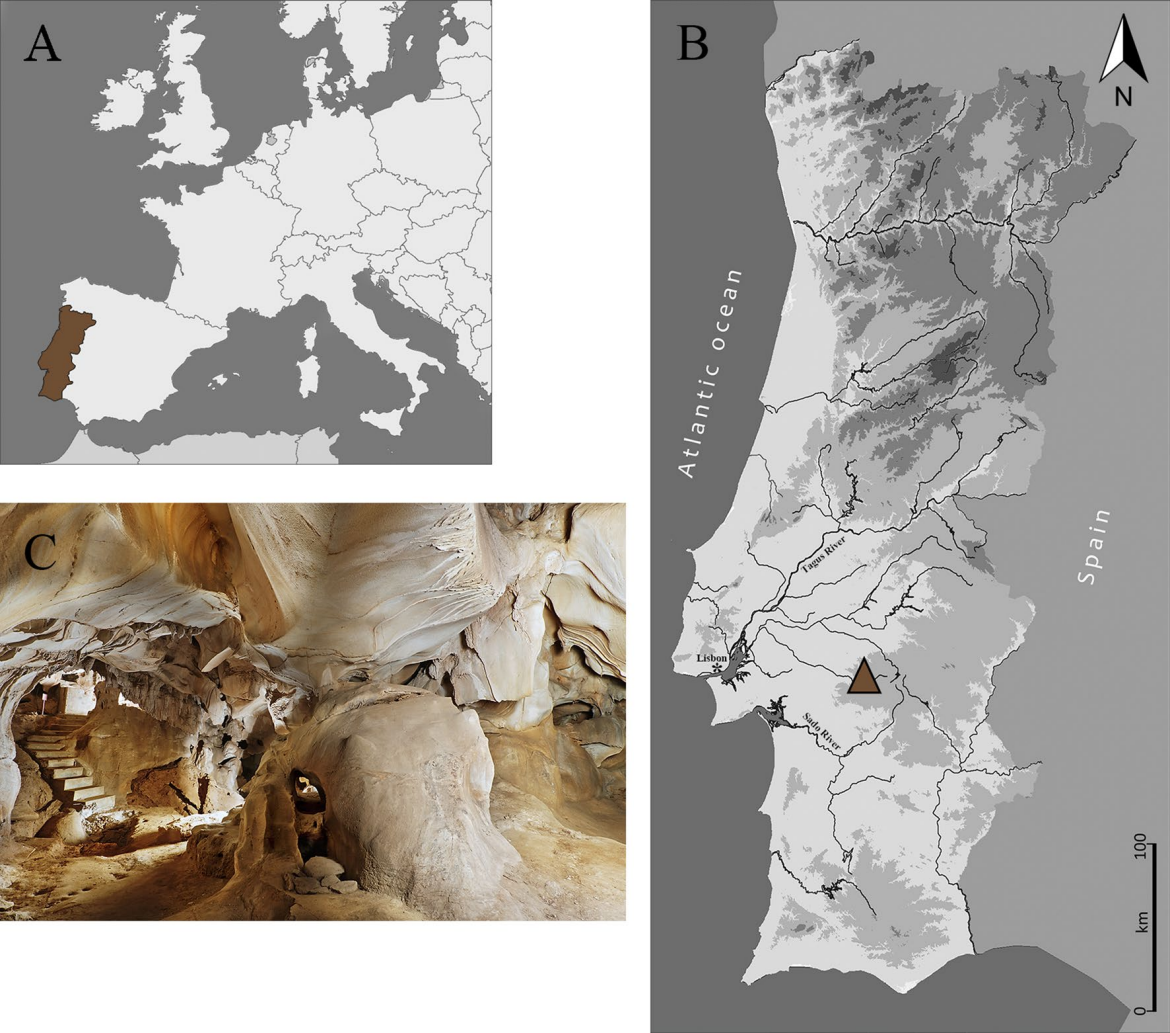
Location of the Escoural Cave in the Alentejo region, southern Portugal (**A**,**B**) and photo of its main room I (**C**) (© M. Ribeiro|DRCA).

To date, the Escoural Cave is the only karst system known in the inner Alentejo (Montemor-o-Novo, southern Portugal) (Fig. 1). It documents archaeological remains from the Middle Palaeolithic (short-term hunting camp), the Upper Palaeolithic (rock art) and the Neolithic (necropolis)^68^. A fortified Chalcolithic settlement was established above Neolithic rock carvings on the top of the hill where the entrance of the cave is. The latter is also surrounded by numerous megalithic monuments (it is in fact the richest region in the country in megalithic architecture), although most of them have not preserved human remains due to the acidity of the granite soils that characterizes the region^69,70^. Additionally, the excavation strategies carried out in those monuments during the 19th and the early twentieth centuries were not the most adequate, especially with regard to the recovery and recording of materials^45,70–72^. Owing to the cultural affinities between cave-necropolis and those monuments^68,73^, the same people most probably used the Escoural Cave and built the megalithic monuments. Therefore, Escoural appears to be the best chance of achieving more comprehensive biocultural knowledge of these somewhat cryptic megalithic builders.

**Figure 2:**
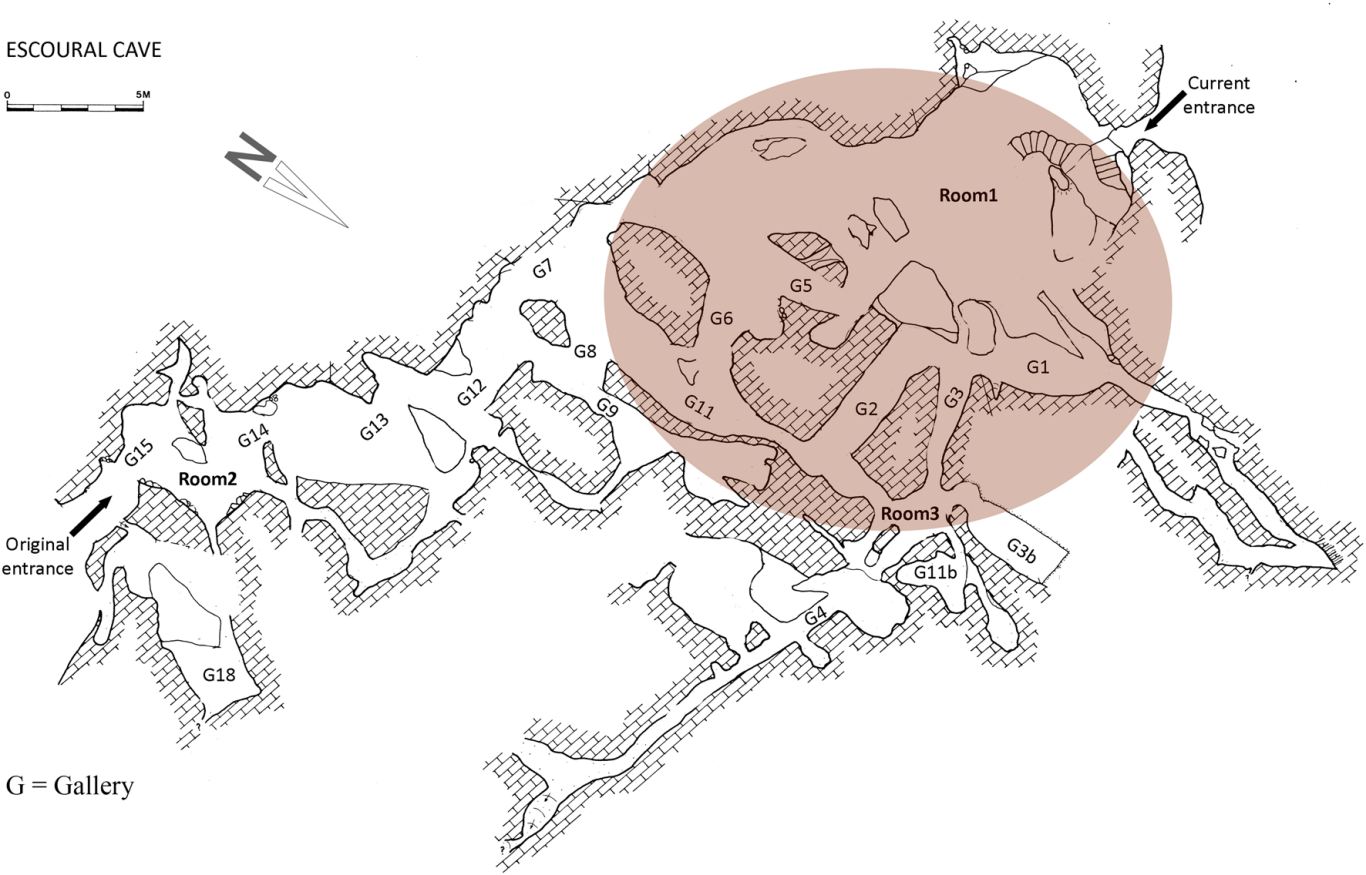
Escoural Cave, plan of the intermediate floor used for funerary purposes. The colored circle shows the area of greatest concentration of human remains. G = Gallery (© P. Lacroix under the responsibility of Ana Cristina Araújo).

The Escoural Cave is divided into three main floors, with the necropolis occupying the intermediate and largest one (Fig. 2) (see Supplementary data S1 for historical details). This floor includes three rooms and several galleries. The main room (Room 1) has access from the current entrance; Room 2 is located on the opposite side, near the original entrance; Room 3 is located at the confluence of galleries 11 and 3 (Fig. 2). Human remains were exposed on the surface of the main room and adjacent galleries, covered by a thick layer of calcite carbonate and associated with diverse grave goods (pottery, polished stones, bone tools, adornments, etc.)^68^.

**Figure 3:**
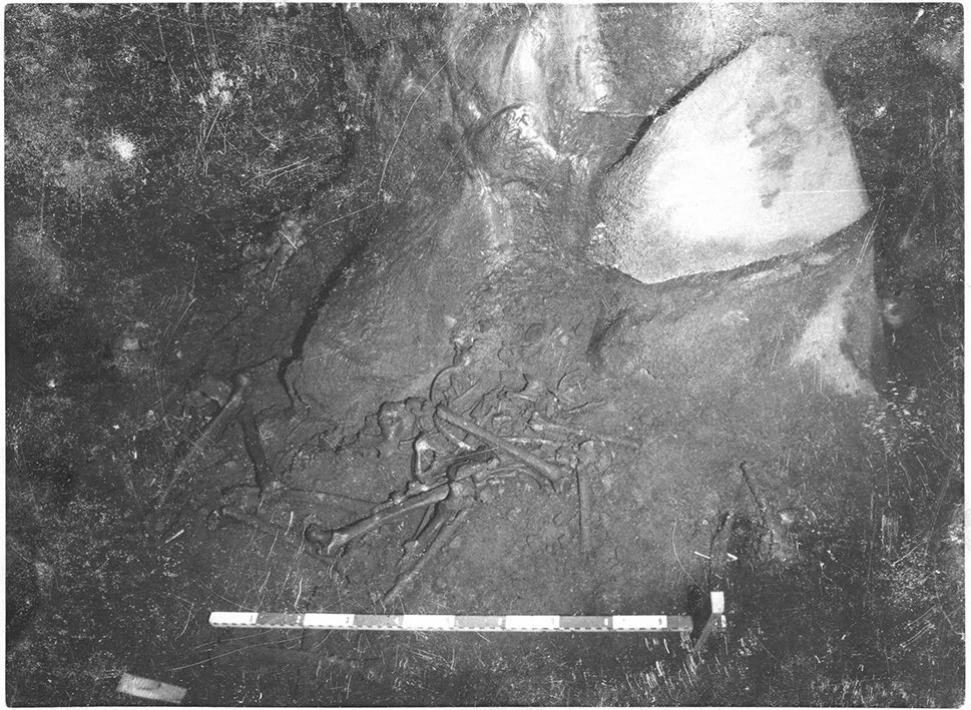
Escoural Cave, Group 7 from Room 1 photographed in the 1960’s. PT/MNA/APMH/2/11/32/18-49 ©DGPC/MNA—Farinha dos Santos/Museu Nacional de Arqueologia/National Museum of Archaeology, Lisbon, Portugal.

Generally, the human remains were commingled on the surface layers and were clustered in “groups” (Fig. 3), attaining a MNI of 109 (42 immatures and 67 matures). Apparently, bones from each individual were not scattered onto multiple clusters, since bone pairs were always found intra-group.

**Figure 4:**
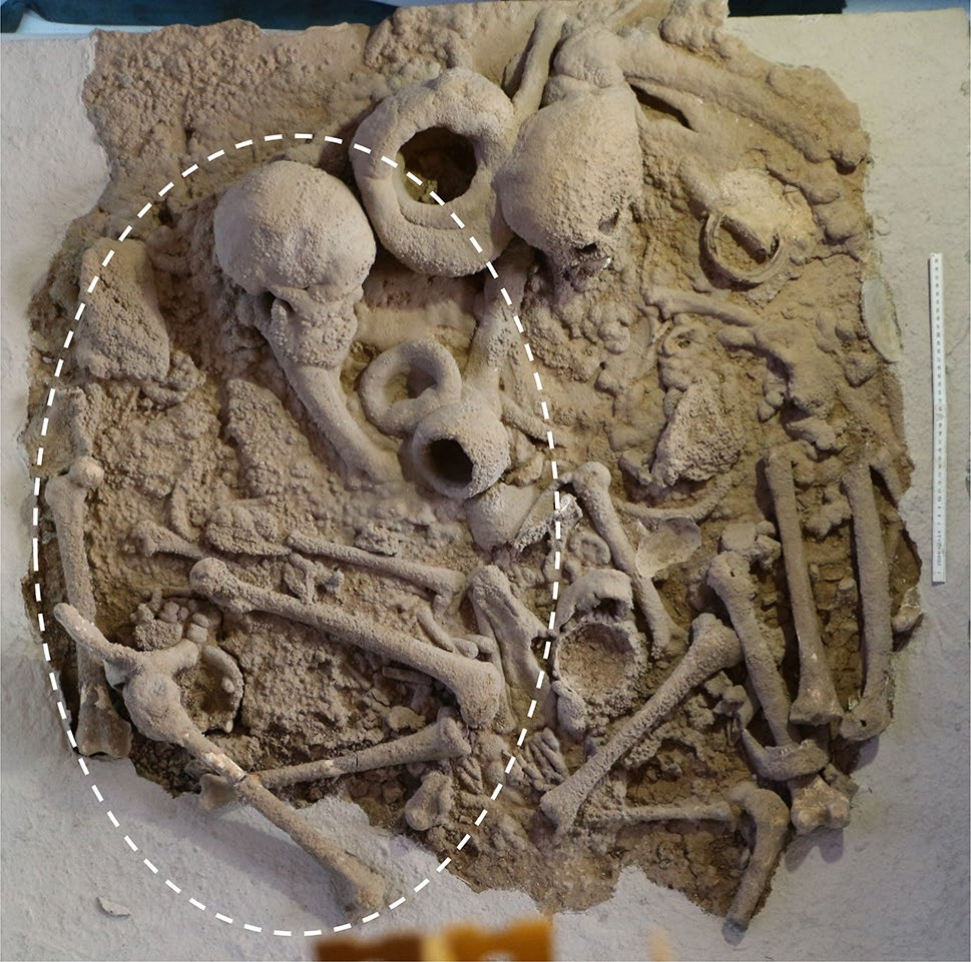
Escoural Cave, Group 1 from Room 1 (the white circle shows an individual in anatomical connection) housed in National Museum of Archaeology in Lisbon, Portugal (© Raquel Granja).

**Figure 5:**
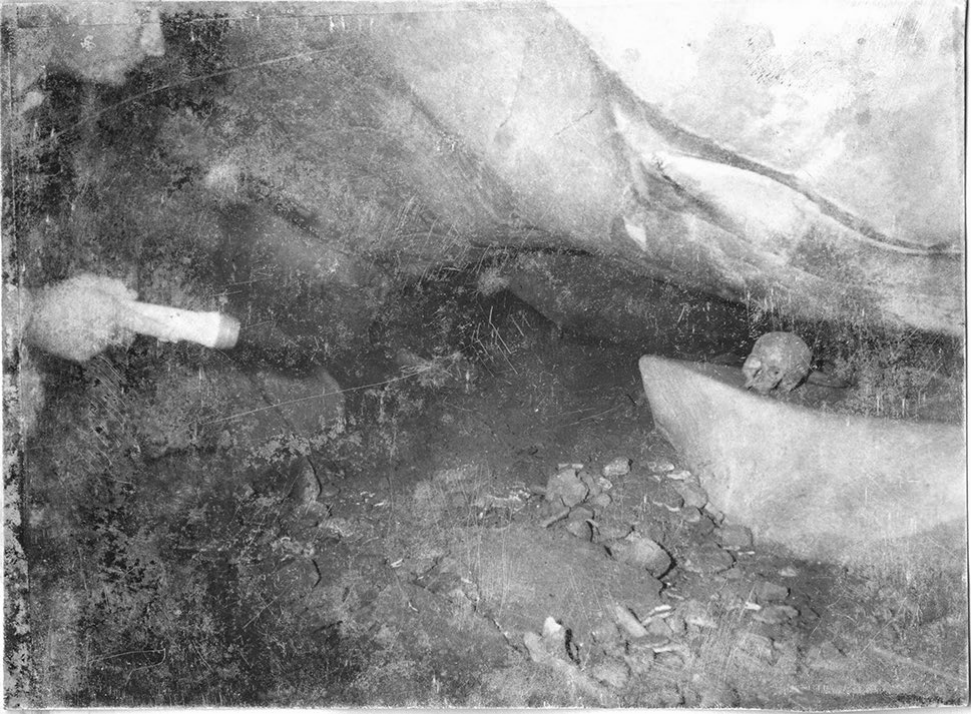
Escoural Cave, cranium on a *shelf* in Gallery 7 photographed in the 1960’s. PT/MNA/APMH/2/11/32/21-49 ©DGPC/MNA—Farinha dos Santos/Museu Nacional de Arqueologia/National Museum of Archaeology, Lisbon, Portugal.

**Figure 6:**
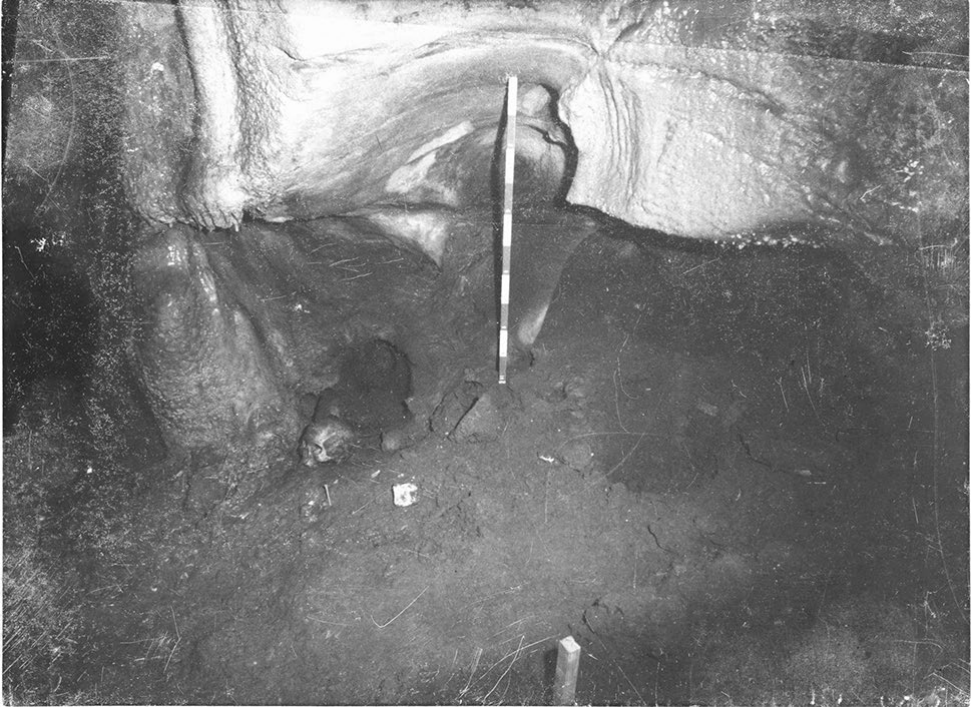
Escoural Cave, cranium in a niche in Room 1 photographed in 1960’s. PT/MNA/APMH/2/11/32/30-49 ©DGPC/MNA—Farinha dos Santos/Museu Nacional de Arqueologia/National Museum of Archaeology, Lisbon, Portugal.

Exceptionally, an adult female individual in partial anatomical connection was present in Group 1 (Fig. 4). She was deposited at the surface on her left flank, with the legs flexed and associated to three ceramic bowls and a probable female cranium, also on her left side. This cranium was not in anatomical connection, being unclear if it belonged to a primary or a secondary deposition. Both depositions were surrounded by several secondarily deposited immature and mature bones. Other two exceptions to the secondary surface depositions comprised apparent secondary depositions of crania in shelves and niches (Figs. 5 and 6).

Santos^74,75^ also mentions an inhumation in a pit, but there is no graphic or photographic documentation of this putative funerary practice in the archives of the National Museum of Archaeology. Human remains were found also outside the cave, next to the south-western natural entrance. Radiocarbon results from bone samples retrieved in this outer area do not differ from those obtained inside the cave, pointing to its funerary use during the Late Neolithic (~ 3600 to ~ 3000 cal BC). Moreover, some skeletal remains display on their surfaces the same carbonate concretions that characterize the material from inside the cave, suggesting that their primary origin was also most likely this same. Therefore, both samples from inside and outside the cave were brought together and studied as one for the purpose of this study.

## Material and methods

### Peptide analysis

Sex estimation through peptide analysis was carried out in 36 lower left permanent canines (LLPCs), for which enamel mineralization occurs at 1.5–6.5 years^65^. Three of these canines were not completely formed (root mineralization was ¼ complete in two teeth while initial root formation with diverse edges was presented by another tooth), eight had a broken root and 25 were mature or in mature mandibles.

Enamel fragments (ca. 10 mg) were washed with MilliQ using an ultrasonic bath and leached for a few minutes using 5% HCl. Then, enamel peptides were extracted submerging enamel fragments in 250 µl of 5% HCl for 1 h. The supernatant was collected and purified through C_18_ in-house stage tips. Resin-bounded peptides were eluted using 50 μL of 60% acetonitrile in 0.1% formic acid. The whole laboratory protocol was performed at the Proteomic facility of the BONES Lab (University of Bologna). Extracted peptides were dried and resuspended in a mixture of water:acetonitrile:formic acid 95:3:2, before the nanoLC-MS/MS measures. Analyses were conducted using Nano UHPLC Ultimate 3000 coupled to an Exploris™ 480 Hybrid Quadrupole-Orbitrap™ Mass Spectrometer via an EASY-Spray source interface, housed at the Centro Interdipartimentale Grandi Strumenti of the University of Modena and Reggio Emilia^76^. Peptides were loaded onto a 300 µm I.D. × 5 mm cartridge, packed with Acclaim PepMap100 C18, 5 µm, 100 Å beads then separated on a 75 μm I.D. × 150 mm EASY-Spray column, packed with Acclaim PepMap100 C18, 3 µm, 100 Å beads. The nanoLC-MS/MS system was controlled with Standard Instrument Integration (SII) for Xcalibur software. All hardware and software for data acquisition were from Thermo Fisher Scientific. The sample (2 µL) was injected onto the precolumn cartridge kept 30 °C using a 10 µL/min flow of a 0.1% formic acid in water solution. The loading step was performed for 2 min then the pre-concentrated peptides were directed toward the Easy-spray column for separation using a reverse 900 nL/min flow at the starting gradient composition. The mobile phase components were (A) 0.1% formic acid in water and (B) 0.1% formic acid in acetonitrile:water (97:3 v/v). Chromatographic separation was performed at 40 °C with B% kept constant at 4% for 3′, then linearly increased from 4 to 26% in 38 min and again from 26 to 50% in 4’; B% was then brought to 95% in 1 min and kept at 95% B for 3 min, before the reconditioning step. Each sample required a total run time of 65 min. The electrospray source was operated in positive ionization mode with the ion transfer tube temperature at 275 °C and a spray voltage of 1600 V. Mass spectrometric detection of peptides was performed using a Data-Dependent Acquisition strategy. An inclusion list with the main endogenous enamel peptides was also included in the method i.e., SM(ox)IRPPY (AMELY; [M+2H]^2+^ 440.2233 m/z); SYEVLTPLK (AMELX,Y; [M+2H]^2+^ 525.2975 m/z), SIRPPYPSY (AMELX; [M+2H]^2+^ 540.2796 m/z); PYFGYFGYH (ENAM; [M+2H]^2+^ 575.7533 m/z); YEVLTPLKWY (AMELX,Y; [M+2H]^2+^ 656.3528 m/z)^54,55^. Ion chromatograms were manually inspected using Xcalibur™ (Thermo Scientific) searching for specific AMELX (amelogenin X isoform 1; *H. sapiens* Q99217) and AMELY (amelogenin Y isoform 2; *H. sapiens* Q99218) peptides, following Stewart et al*.*^58^ and Lugli et al*.*^54^ for sex estimation. No AMEL-peaks were detected in the extraction blank, processed and analysed along with the samples. Raw chromatograms (.raw) are deposited in Zenodo (https://zenodo.org/record/8188927).

### Linear enamel hypoplasias analysis

All 36 lower left permanent canines were macroscopically examined for the presence of LEH. When present, the distance between the LEH and the cementum-enamel junction (CEJ) was measured with a dental caliper. To estimate the age at which these stressful episodes occurred, the recommendations of Cares-Henriquez and Oxenham^77^ and Cares-Henriquez^78^ were adopted whenever the occlusal wear was equal or inferior to grade 2 using the classification from Smith^79^ adapted by Silva^80^. The wear was described as 10% if the tooth presented a grade-2 wear, as 5% if it presented a grade-1 wear and 0 if no wear was present.

### Odontometric analysis

The buccolingual diameter was measured in the 36 LLPCs and the work of Cardoso^27^ was used as reference (sex discriminating cut-off point: 7.73 mm) for odontometric sex estimation. Measurements were taken three times and only the median value was used for subsequent analyses. The intra- and inter-observer variations were calculated based on the measurements obtained by two researchers (RG & DG). In the case of the intra-observer variation, measurements were obtained on two different moments with a one-year interval. Observer variation was obtained by calculating the relative technical error of measurement (%TEM)^81,82^.

### Statistical analysis

Sex differences regarding the occurrence of LEH were tested using the Chi-square statistic. Sex differences in mean LEH age of onset were tested using an unpaired Student’s t-test. The agreement between sex classifications obtained through peptide analyses vs odontometric analyses was tested using the Kappa Cohen statistics. The intra– and inter-observer variations of the buccolingual diameter of the lower left permanent canine were calculated using the technical error of measurement on a sample of 25 specimens^81,82^.

## Results

### Sex profile through peptide analysis

**Figure 7:**
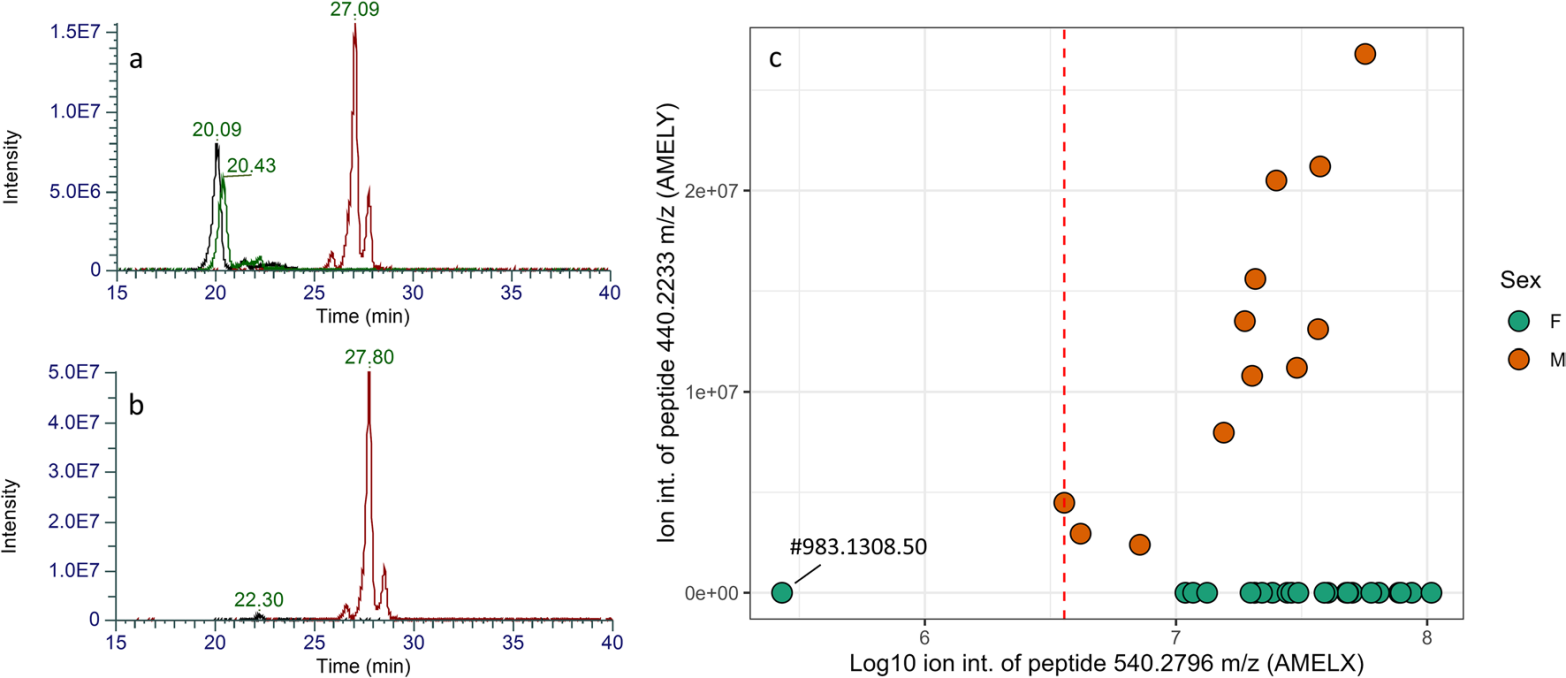
Ion chromatograms of individuals estimated as (**a**) male (GDE 3, nº 984.98.266) and (**b**) female (GDE 2, nº 983.340.45). Chromatograms search was performed using Xcalibur software (Thermo Scientific) with a mass tolerance of 5 ppm. The black peak is peptide SM(ox)IRPPY (AMELY; [M+2H]^2+^ 440.2233 m/z); the green peak is peptide M(ox)IRPPY (AMELY; [M+2H]^2+^ 396.7073 m/z), while the red peak is peptide SIRPPYPSY (AMELX; [M+2H]^2+^ 540.2796 m/z); retention times are as expected (see, Lugli et al*.*^54^). (**c**) Maximum ion intensities of peptide SM(ox)IRPPY (AMELY; [M+2H]^2+^ 440.2233 m/z) vs peptide SIRPPYPSY (AMELX; [M+2H]^2+^ 540.2796 m/z), this latter reported as log10; males (as estimated through proteomics) are orange dots while (possibly) females are dark-green dots; the red dashed line is the male with the lowest 540.2796 m/z signal.

Proteomics results are presented in Table 1 (see Supplementary Table S1 for details) and examples of male and female chromatograms are given in Fig. 7a and b. Sex estimation was possible in all cases, i.e. amelogenin peptides were present in all the enamel samples. 12 out of 36 samples showed the presence of AMELY specific peptides, indicating unambiguously male sex. On the other hand, 23 individuals were identified as likely females, because of the lack of AMELY and high ion signals of AMELX (~ 10^6^–10^7^). One sample from inside the cave (Room 1), likely due to taphonomic alterations, resulted in lower spectrum quality and ion intensity (i.e. AMELX 540.2796 m/z signal ~ 10^5^), hampering a confident sex estimation. Since the absence of AMELY_HUMAN can be due to the female sex, low-resulting signals of AMELY peptides, or AMELY deletion^83^, we prefer to treat the result of sample nº 983.1308.50 with caution, being AMELX-peptide ~ 100 times lower in signal compared to the other individuals (see Fig. 7c).

**Table 1:** Escoural Cave sex profile of 36 individuals, based on peptide analysis.

	M	F	F?	M:F
Inside cave	8	21	1	0.38:1
Outside cave	4	2	0	2.00:1
Total	12	23	1	0.52:1

Based on this result, the overall sex ratio of 0.52:1 in favour of females. Bearing in mind that the sample outside the cave was relatively small, a higher number of males was observed outside the cave compared to females. A more complete sex profile of the assemblage was obtained by adding one other female individual whose sex estimation was achieved through the hip bone morphology of the individual in anatomical connection. As a result, a more female-prone sex ratio of 0.5:1 (M:F) was obtained overall.

### Linear enamel hypoplasias

The majority of the individuals (24/36, 67%) present at least one enamel defect, a trend observed in both females (15/23, 65%) and males (9/12, 75%) with no significant sexual differences found between both sexes (χ^2^ = 350; *p* = 0.554). The probable female, who did not present any LEH, was not included in this analysis. Most individuals displaying only one LEH defect (females: 9/15, 60%; males: 6/12, 50%). Two defects were observed in 27% of females (4/15) and 17% of males (2/12) while three defects were observed in 13% of females (2/15) and 8% of males (1/12).

The mean LEH age of onset was 3.5 years in 12 females and 3.9 years in 7 males, the difference being non-significant (*p* = 0.116). The youngest age of onset was 2.6 years in females and 3.2 years in males while the oldest age of onset was 4.4 years and 4.6 years, respectively. In both sexes, the highest number of LEH cases occurred during the third year of life.

### LEH frequency per sex

If the LEH frequency is analysed by sex, it is possible to conclude that there are no significant differences between sexes (*p* = 0.554). Apparently, both sexes were equally affected by physiological stress events.

### Odontometric sex estimation

The intra- and inter observer variations of the buccolingual diameter provided %TEM below 2%, demonstrating the good replicability and repeatability of this standard measurement (see Supplementary Table S2 for details). The high coefficient of reliability (≥ 93%) indicated that only a small portion of the measurement variance in the sample was the result of measurement error.

Assuming that peptide-based sex estimations are correct, the reliability of odontometric sex estimation was calculated. The buccolingual diameter allowed for 80% correct sex classification of the overall sample. Based on the Kappa Cohen statistics, the agreement between the peptide and the odontometric sex estimations was moderate (0.53; *p* = 0.001) and 91.3% of females (21/23) and only 58.3% of males (7/12) were allocated to the correct sex.

## Discussion

### Sex profile

In human societies, natural sex ratios usually range from about 0.95:1 to 1.02:1 (M:F), with exceptions being reported in cases such as heavy warfare losses or intense immigration^84^. Sex ratios of ≤ 0.9:1 and ≥ 1.05:1 (M:F) are considered to be extreme values^84^. Therefore, the sex ratio observed in the Escoural Cave (0.5:1, M:F) can be assumed to have been extremely unbalanced in favour of females and, most probably, the reason for that pattern cannot be attributed to natural dynamics. The Escoural Cave was almost totally excavated, so the unbalanced sex ratio is probably not the result of excavation-related bias either (e.g., differential sieving procedures). On the other hand, differential sex-related preservation of skeletal elements does not seem to explain it as well since that usually benefits male representation due to their more robust physique^85^. It should be noted that all but one of the male teeth presented dental wear incompatible with a non-adult age.

Therefore, the well-marked imbalance in the sexual profile of the dead deposited in the Escoural Cave seems to be more related to anthropogenic choices of a socio-cultural nature and may reflect: (i) the contemporary demographic pattern, also unbalanced or (ii) choices dictated by cultural perceptions regarding the dead and death.

In contrast to the Escoural Cave, a more sex-balanced ratio ranging from 0.96:1 to 0.9:1 (M:F) was proposed for the Bom Santo cave, located in the Estremadura region and also used as a necropolis, but during the Middle Neolithic (3800–3400 cal BC)^4,86^. However, in the case of this funerary cave, sex-ratio was exclusively obtained through odontometric evaluation; so, no definitive conclusions can be obtained.

Other Portuguese cave-necropolises offer little data in terms of sex-ratio. For instance, most were used not only during the Early, Middle and Late Neolithic, but also during the Copper, Bronze and Iron ages, making the analysis even more difficult. Additionally, the number of individuals on which sex estimation was achieved is much lower than the minimum number of individuals (MNI) recorded in each of these cave-contexts. This problem becomes even more complex if the discrepancy in the assessment methods used for sex estimation (and not always fully available) is added to the debate. Consequently, the set of cave-necropolises recorded for Portuguese archaeology does not meet the conditions to support the hypothetical sex-ratio imbalance suggested by the data from the Escoural Cave. Its absolute chronology, based on several radiocarbon results (more than 30), points to their use mainly during the Late Neolithic (~ 3600 to  ~ 3000 cal BC), although three additional values deviate from this chrono-cultural framework (this subject deserves a much deeper discussion, which does not fit into this paper). Once again, Bom Santo remains the best reference for comparisons, although it is somewhat older than Escoural. To reinforce this view, both have very similar material cultures.

Cases of very unbalanced sex ratios in caves have also been reported for other parts of the Iberian Peninsula. Fernández-Crespo and de-la-Rúa^87^ studied the sex profile of Late Neolithic past populations from the northern Spain and found sex ratios varying from 1.1:1 to 2:1 (M:F) in dolmens, whereas the caves yielded ratios ranging from 0.08:1 to 0.5:1 (M:F). It was argued that cultural factors may have played an important role in the origin of the bias^87^. Unfortunately, due to preservation issues, Portuguese dolmens usually have small amounts of human bones thus it is difficult to investigate potential unbalanced sex ratios^88–91^. Amelogenin sex estimation may help circumventing this obstacle since dental remains tend to preserve better and even allow higher MNI in some cases^7,44^.

On the other hand, Cintas-Peña and Sanjuán^92^ and Cintas-Peña and Herrero-Corral^93^ detected a higher proportion of male (or possible male) over female (or possible female) individuals in Iberian Neolithic assemblages (n = 119 –[23.11%] vs 79 –[15.34%]). However, the large proportion of individuals with indeterminate sex (n = 207 [61.56%]) prevents reliable conclusions. Moreover, these authors used several types of sepulchres on their analysis, with a large predominance of pit necropolises. In her study of seven Late Neolithic/Chalcolithic collective tombs (including dolmens, tholos, natural and artificial caves), Silva^6,7^ also detected a higher proportion of females, based on metric analyses of femurs, humeri, calcanei and tali. However, these studies suffer from the issues mentioned above and/or are from later chronologies.

As for hypothetical scenarios explaining the unbalanced sex profile in the Escoural Cave, no archaeological evidence is particularly instructive. We were unable to build a comprehensive age and health profile for this assemblage, so it was not possible to assess if the male population was particularly old or unhealthy compared to females. If so, this could be suggestive of a scenario in which younger, healthier, or more active men were dying somewhere distant from the cave (and the settlement). This would possibly be the case in situations involving warfare or transhumance, assuming that those activities were mainly performed by males, which is not at all clear. So far, no strontium isotope analyses were performed to check for possible migration patterns, which could have contributed to the sex profile here observed.

Apparently, the Escoural Cave was not restricted to one of the sexes since both female and male individuals were found in it. However, the possibility of a sex restriction rule having occurred at some point of its utilization as a necropolis cannot be entirely discarded. Such occurrence, even if implemented during a shorter period could result in the unbalanced sex profile obtained by our forcibly synchronic analysis. If a sex restriction was in place at some point in time, this would mean that specific funerary ordinances were applied to segments of the community. As mentioned before, the dolmens in the region did not preserve, in most cases, organic material that can be used for comparative purposes.

### LEH

The Escoural assemblage presented an unusually high frequency of LEH in both female and male individuals. That becomes clear from both diachronic and synchronic comparisons with other prehistoric necropolises in the Portuguese territory reflecting a wide chronology from the 7th to the 3th millennia cal BC. Table 2 provide chrono-culturally homogeneous sites for which LEH observations per individual were possible.

**Table 2:** LEH frequencies in prehistoric Portuguese necropolises.

Site (reference)	Type	Chronology	MNI	Skeletal piece	N	LEH Frequency
		cal BC				N (%)
Moita do Sebastião (1)	Open air	6360–5350	85	Skull	20	14 (70)
Cabeço da Arruda (2)	Open air	6270–4840	110	Skull	64	26 (41)
Cova da Onça (3)	Open air	5880–5000	32	Skull	21	4 (19)
Cabeço da Amoreira (4)	Open air	5830–4720	29	Skull	7	2 (29)
Caldeirão (5)	Cave	5470–3540	16	Tooth 33	4	3 (75)
Escoural (6)	Cave	4238–2626	109	Tooth 33	36	24 (67)
Sobreira I (7)	Dolmen	3640–3380	6	Permanent teeth	5*/21	2 (33,3)
Carrascal (8)	Dolmen	3650–3350	14	Permanent teeth	8*/97	3 (30)
Ansião (9)	Dolmen	3637–3094	37	Tooth 22	13	2 (15)
Pedras Grandes (10)	Dolmen	3510–3100	13	Permanent teeth	13*/55	3 (23)
Perdigões, Tomb I (11)	Tholos	2870–2460	103	Tooth 13 & 43	43 & 24	7 (16) & 10 (42)
Perdigões, Tomb II, Chamber (12)	Tholos	2860–2200	30	Permanent teeth	2*/266	1 (0,4)
Perdigões, Tomb II, Atrium (13)	Tholos	2860–2200	26	Permanent teeth	3*/212	1 (05)

MNI: minimum number of individuals; N: number of individuals which allowed LEH observation; LEH frequency (N): number of individuals (skeletons) with LEH; *: number of teeth with LEH. References (^dd^—dating data, ^ad^—anthropological data): (1)^94^^ad,^^95^^dd,^^96^^dd,^^97^^dd,^^98^^dd,^^99^^dd^. (2)^94^^ad,^^99^^dd,^^100^^dd;^^101^^dd,^^102^^dd^. (3)^94^^ad,^^97^^dd^. (4)^94^^ad,^^97^^dd^. (5)^103^^ad^,^104^^dd^. (6) Unpublished (7)^71^^dd, ad^. (8)^44^^dd,^^90^^ad^. (9)^7^^dd, ad^. (10)^44^^dd,^^91^^ad^. (11)^2^^ad^. (12)^8^^ad,^^105^^dd^. (13)^8^^ad,^^105^^dd^.

The LEH frequency in the Portuguese prehistoric necropolises is below 42% in most of the sites. The ones with the highest LEH frequencies (above 60%) are also the ones where fewer individuals were assessed (below 40% of the MNI), so interpretation must be cautious.

Regarding Neolithic caves (Escoural and Caldeirão), LEH was observed in more than two thirds of the individuals. On the other hand, these frequencies appeared all to be under or equal to 30% in dolmens. However, due to the scarcity of data is not possible, for now, to draw inferences.

Regarding the Escoural’s LEH onset ages, values are very close to the weaning end age (3.3 ± 0.7 years old) estimated through isotopes by Fernández-Crespo et al.^106^ in several Spanish Late Neolithic cave necropolises. However, in the case of Escoural, it was not possible to estimate the age of 16 individuals because teeth presented a wear grade higher than 2^79,80^, thus precluding the estimation of LEH onset age.

### Odontometric sex estimation

The canine buccolingual diameter to estimate the sex of the individuals from the Escoural Cave was somewhat reliable. It allowed correct classification of four in every five individuals, even though the reference cut-off point used for that purpose was obtained from a recent Portuguese population who lived five millennia after the Escoural population^27^. Therefore, metric differences due to secular trend and population variability are to be expected as deduced from the very variable odontometric profiles of varied populations^27,107–113^. Indeed, although sex estimation accuracy was high, it was relatively unbalanced between the two sexes thus suggesting that the reference sex discriminating cut-off point (7.73 mm) was not entirely adjusted to the Escoural assemblage. Looking at the average canine buccolingual diameter in the latter (sex pooled mean = 7.54 mm; weighed sex pooled mean = 7.43 mm), it became clear that the reference recent population presented larger mean canine metric features. As a result, the reference cut-off point led to the high number of males mistakenly attributed to the female sex. The Escoural sex pooled mean could have been used as a sample-specific sex discriminating cut-off point, an approach used previously in archaeological research^4,114^ but we chose not to do it because the required sample was smaller than the 40 specimens recommended by Albanese et al.^31^. Only used here as an illustration of the benefit of using sample-specific metric references that are more adjusted to the population under study, the informal testing of the sample-specific cut-off point would result in a more balanced correct sex classification (females: 77%; males: 75%).

As expected, the odontometric approach does not guarantee a reconstitution of the sex profile as reliable as those obtained from DNA and peptide analyses. Some degree of error should be expected from sex ratios obtained through canine buccolingual diameters. 20 to 25% of the sample was incorrectly sex estimated following the application of sample-specific cut-off points in the Escoural assemblage. Therefore, the use of intervals taking into account such magnitudes of variation appears to be recommendable.

## Conclusion

This research sheds new light on the way of life of the Neolithic community that used the Escoural Cave 5000 years ago. To date, this is the largest Portuguese sample subjected to peptide sex estimation. Possibly, the cave played a differential role in the funerary processing of women and men of this Neolithic community which mirrors the one reported for communities from northern Iberia. Nonetheless, the LEH analysis suggested that stress physiological episodes in the infancy were probably equally frequent and transverse to both sexes. The large presence of LEH can possibly be explained by a stressful weaning process which equally affected all analysed individuals at similar ages independently of their sex.

The new and important data gathered in this research were only possible to obtain thanks to the uniquely good preservation of the numerous human remains of this archaeological site which is culturally homogenous. Moreover, the large number of recovered teeth enabled the proteomic analysis of sex-specific amelogenin peptides. This new methodology is ground-breaking and overcomes problematic issues related to the sex estimation of immature and mature individuals, especially in contexts where remains are commingled, scattered, and/or fragmented, as is often the case in prehistorical necropolises. Peptide analysis allowed us to go further in terms of a more holistic assessment of funerary practices, demographic data or even pathologies. Therefore, this approach can promote the active re-visitation of other emblematic Portuguese Neolithic sites and it would be interesting to extend this approach to the individuals deposited in other coeval tombs, as dolmens and caves, to replicate the study presented here.

Peptide analysis appears to have an advantage over the forcibly reductive osteological examination of commingled and scattered remains as well as over the considerably more expensive aDNA analysis thus constituting an appealing resource for bioarchaeological research.

### Supplementary Information

Supplementary Information.

Supplementary Table S1.

Supplementary Table S2.

## Data Availability

The generated during and/or analysed during the current study are available in the Zenodo repository (https://zenodo.org/record/8188927).
