# Supplementary material for: Unbalanced sex-ratio in the Neolithic individuals from the Escoural Cave (Montemor-o-Novo, Portugal) revealed by peptide analysis
Source: Sci Rep. 2023 Nov 14;13:19902. doi: 10.1038/s41598-023-47037-4 (PMC10646114; doi:10.1038/s41598-023-47037-4)
Supplement: Supplementary file 1 — Supplementary Information. [file 41598_2023_47037_MOESM1_ESM.pdf]

Title: Unbalanced sex-ratio in the Neolithic individuals from the Escoural Cave (Montemor-o-Novo, Portugal) revealed by peptide analysis

Raquel Granja, Ana Cristina Araújo, Federico Lugli, Sara Silvestrini, Ana Maria Silva, David Gonçalves

Short Outline of the Archaeological Interventions to the Escoural Cave

The Escoural Cave was discovered in 1963 during stone mining works and was the subject of archaeological excavations between 1963 and 1968 by Farinha dos Santos, archaeologist and assistant of Manuel Heleno, the former director of the National Museum of Archaeology in Lisbon. Later, between 1989 and 1992, the site was the subject of new investigations by a Portuguese-Belgian team with the main purpose of i) studying the neolithic material associated with the necropolis, clarifying its stratigraphic context [1] and ii) assessing the archaeological potential of the cave for preserving Palaeolithic occupations by opening test-pits inside and outside the cave [2]. In 2018, the MORESCOURAL interdisciplinary project was implemented to study the Escoural Cave in terms of osteoarchaeology, genomics, palaeodiet and subsistence, mobility, funerary practices, material culture, and chronological framework.

References

[1] Araújo, A. C. & Lejeune, M. *Gruta do Escoural: necrópole neolítica e arte rupestre paleolítica* (Trabalhos de Arqueologia 8, Instituto Português do Património Arquitectónico e Arqueológico, 1995).

[2] Otte, M. & Silva, A. C. *Recherches préhistoriques à la Grotte d'Escoural, Portugal* (ERAUL 65, Université Liège, 1996).
