# Supplementary material for: Unbalanced sex-ratio in the Neolithic individuals from the Escoural Cave (Montemor-o-Novo, Portugal) revealed by peptide analysis
Source: Sci Rep. 2023 Nov 14;13:19902. doi: 10.1038/s41598-023-47037-4 (PMC10646114; doi:10.1038/s41598-023-47037-4)
Supplement: Supplementary file 2 — Supplementary Table S1. [file 41598_2023_47037_MOESM2_ESM.pdf]

Title: Unbalanced sex-ratio in the Neolithic individuals from the Escoural Cave (Montemor-o-Novo, Portugal) revealed by peptide analysis

Raquel Granja, Ana Cristina Araújo, Federico Lugli, Sara Silvestrini, Ana Maria Silva, David Gonçalves

Supplementary Table S1 – Age at death and sex estimations for each of the tooth 33 analysed, and LEH frequencies and age of occurrence. Peptides (m/z) are reported as maximum ion intensities.

		**Peptides**				**Buccolingual diameter**				**LEH**	
**Individual**	**Age at death (years)** [1]	***m/z* 540.2796**	***m/z* 440.2233**	***m/z* 396.7073**	**Peptide sex estimation**	**Obs 1 (mm)**	**Sex (Obs 1)** [2]	**Obs 2 (mm)**	**Sex (Obs 2)** [2]	**Frequency**	**Age of occurrence (years)** [3, 4]
983.333.19	≥ 12,5	2.06E7	.	.	F	7,10	F	7,20	F	0	
983.333.20	≥ 12,5	2.42E7	.	.	F	6,65	F	6,67	F	2**	
983.340.45	6,5-9,5	5.03E7	.	.	F	7,04	F	7,05	F	1	4,42
983.362.116	≥ 12,5	2.78E7	.	.	F	6,74	F	6,87	F	0	
983.371.29	.	8.68E7	.	.	F	7,87	<u>**M**</u>	7,97	<u>**M**</u>	0	
983.371.30	.	4.77E7	.	.	F	7,39	F	7,46	F	3	3,05; 3,76; 4,22
983.376.31	.	2.05E7	.	.	F	7,61	F	7,52	F	0	
983.383.7	4,5-7,5	1.04E8	.	.	F	6,73	F	6,86	F	3	
983.386.112	≥ 12,5	2.20E7	.	.	F	7,22	F	7,26	F	0	
983.1299.104	≥ 12,5	7.73E7	.	.	F	6,92	F	6,90	F	1	3,90
983.1301.78	≥ 16,5	2.88E7	.	.	F	7,06	F	7,09	F	1	2,98
983.1306.53	6,5-9,5	7.85E7	.	.	F	7,77	<u>**M**</u>	7,80	<u>**M**</u>	0	
983.1348.91	≥ 20,5	4.85E7	.	.	F	6,79	F	6,00	F	1	3,65
984.171.165	≥ 10,5	6.43E7	.	.	F	6,92	F	6,95	F	1	3,43
984.171.169	.	4.83E7	.	.	F	7,38	F	7,30	F	1	4,18
984.459.32	≥ 14,5	3.07E7	.	.	F	6,70	F	6,78	F	2	3,30; 4,15
984.461.13	≥ 17,5	1.09E7	.	.	F	7,63	F	7,61	F	2	2,57; 3,57
984.461.14	≥ 20,5	1.98E7	.	.	F	7,28	F	7,57	F	0**	
2006.442.21	≥ 20,5	5.99E7	.	.	F	7,29	F	7,25	F	1	3,60

2006.442.51	≥ 12,5	4.02E7	.	.	F	6,60	F	6,58	F	1	3,86
2022.30.1	≥ 20,5	1.17E7	.	.	F	7,70	F	7,67	F	0	
984.98.267*	≥ 12,5	1.33E7	.	.	F	7,45	F	7,42	F	2**	
2006.307.23*	≥ 12,5	3.90E7	.	.	F	7,34	F	7,32	F	1	3,03
983.1308.50	.	2.70E5	.	.	F?	7,86	**M**	7,92	M	0	
983.1293.5	.	2.07E7	1.56E7	1.04E7	M	8,42	M	8,44	.	2	3,69; 4,42
983.1301.49	.	2.01E7	1.08E7	7.14E6	M	7,43	**F**	7,55	**F**	1	3,96
983.1306.52	≥ 12,5	2.51E7	2.05E7	1.23E7	M	7,85	M	7,82	M	1**	
984.459.30	≥ 20,5	3.03E7	1.12E7	7.66E6	M	7,40	**F**	7,56	**F**	1	4,10
2006.438.4	≥ 20,5	3.59E6	4.48E6	2.77E6	M	8,64	M	8,68	M	0	
2006.442.24	≥ 20,5	3.68E7	1.31E7	7.65E6	M	7,36	**F**	7,58	**F**	0	
2006.442.50	≥ 12,5	7.18E6	2.39E6	1.44E6	M	8,22	M	.	.	2	3,83; 4,09
2006.446.5	≥ 20,5	4.17E6	2.94E6	1.81E6	M	8,43	M	7,87	M	3**	
984.98.264*	≥ 12,5	1.88E7	1.35E7	9.96E6	M	7,42	**F**	7,35	**F**	1	4,55
984.98.266*	≥ 12,5	1.55E7	7.97E6	5.66E6	M	7,67	**F**	7,74	M	0	
984.98.300: 984.167.158*	≥ 20,5	5.67E7	2.68E7	1.90E7	M	8,04	M	8,31	M	1	3,23
984.118.76*	≥ 12,5	3.75E7	2.12E7	1.37E7	M	7,88	M	7,94	M	1	3,86

* - exterior of the cave (all the others come from inside the cave); ** - wear grade higher than 2 (classification from Smith [5] adapted by Silva [6]); bold and underlined – incorrect sex estimates

References

[1] AlQahtani, S. J., Hector, M. P. & Liversidge, H. M. Brief communication: the London atlas of human tooth development and eruption. *Am. J. Phys. Anthropol.* **142**, 481-490 (2010).

[2] Cardoso, H. F. V. Sample-specific (universal) metric approaches for determining the sex of immature human skeletal remains using permanent tooth dimensions. *J. Archaeol. Sci.* **35**, 158-168 (2008).

[3] Cares-Henriquez, A. LEH chronology calculator (Version 1.0). https://www.lehtools.com (2019).

[4] Cares-Henriquez, A. & Oxenham, M. F. New distance-based exponential regression method and equations for estimating the chronology of linear hypoplasia (LEH) defects on the anterior dentition. *Am. J. Phys. Anthropol.* **168**, 510-520 (2018).

[5] Smith, B. H. Patterns of molar wear in hunter-gatherers and agriculturalists. *Am. J. Phys. Anthropol.* **63**, 39-56 (1984).

[6] Silva, A. M. *O Hipogeu de Monte Canelas I (IV – III milénios a.C.): Estudo paleobiológico da população humana exumada* (University of Coimbra, 1996).
