# Supplementary material for: Unbalanced sex-ratio in the Neolithic individuals from the Escoural Cave (Montemor-o-Novo, Portugal) revealed by peptide analysis
Source: Sci Rep. 2023 Nov 14;13:19902. doi: 10.1038/s41598-023-47037-4 (PMC10646114; doi:10.1038/s41598-023-47037-4)
Supplement: Supplementary file 3 — Supplementary Table S2. [file 41598_2023_47037_MOESM3_ESM.pdf]

Title: Unbalanced sex-ratio in the Neolithic individuals from the Escoural Cave (Montemor-o-Novo, Portugal) revealed by peptide analysis

Raquel Granja, Ana Cristina Araújo, Federico Lugli, Sara Silvestrini, Ana Maria Silva, David Gonçalves

Supplementary Table S2 - Escoural Cave, results of the inter and intra-observer variation of the buccolingual diameter of the lower left permanent canine

Inter-observer

Tooth ID	Obs. 1	Obs. 2		Relative difference
983.333.19	7,10	7,20	0,01	1,39
983.333.20	6,65	6,67	0,0004	0,30
983.340.45	7,04	7,05	1E-04	0,14
983.362.116	6,74	6,87	0,0169	1,89
983.371.29	7,87	7,97	0,01	1,25
983.371.30	7,39	7,46	0,0049	0,94
983.376.31	7,61	7,52	0,0081	-1,20
983.383.7	6,73	6,86	0,0169	1,90
983.386.112	7,22	7,26	0,0016	0,55
983.1293.5	8,42	8,44	0,0004	0,24
983.1299.104	6,92	6,90	0,0004	-0,29
983.1301.49	7,43	7,55	0,0144	1,59
983.1301.78	7,06	7,09	0,0009	0,42
983.1306.52	7,85	7,82	0,0009	-0,38
983.1306.53	7,77	7,80	0,0009	0,38
983.1308.50	7,86	7,92	0,0036	0,76

Intra-observer

Tooth ID	Obs. 1	Obs. 1		Relative difference
983.333.19	7,10	7,21	0,0121	1,53
983.333.20	6,62	6,67	0,0025	0,75
983.340.45	7,04	7,04	0	0,00
983.362.116	6,70	6,77	0,0049	1,03
983.371.29	7,84	7,91	0,0049	0,88
983.371.30	7,37	7,42	0,0025	0,67
983.376.31	7,61	7,64	0,0009	0,39
983.383.7	6,65	6,75	0,01	1,48
983.386.112	7,18	7,22	0,0016	0,55
983.1293.5	8,42	8,43	1E-04	0,12
983.1301.49	7,40	7,43	0,0009	0,40
983.1301.78	7,06	6,93	0,0169	-1,88
983.1306.53	7,75	7,83	0,0064	1,02
983.1308.50	7,84	7,88	0,0016	0,51
983.1348.91	6,44	6,81	0,1369	5,43
984.171.165	6,94	6,90	0,0016	-0,58

983.1348.91	6,79	6,00	0,6241	-13,17
984.171.165	6,92	6,95	0,0009	0,43
984.171.169	7,38	7,30	0,0064	-1,10
984.459.30	7,40	7,56	0,0256	2,12
984.459.32	6,70	6,78	0,0064	1,18
984.461.13	7,63	7,61	0,0004	-0,26
984.461.14	7,28	7,57	0,0841	3,83
2006.438.4	8,64	8,68	0,0016	0,46
2006.442.21	7,29	7,25	0,0016	-0,55
			0,145043	TEM
			7,36	Mean
			1,971927	%TEM
			0,021038	TEM^2
			0,544296	σ
			0,296258	σ ^2
			0,071011	TEM^2/ σ ^2
			0,928989	C.RELIABILITY (R)

984.171.169	7,38	7,42	0,0016	0,54
984.459.30	7,40	7,45	0,0025	0,67
984.459.32	6,70	6,71	1E-04	0,15
984.461.13	7,63	7,63	0	0,00
984.461.14	7,30	7,26	0,0016	-0,55
2006.438.4	8,75	8,64	0,0121	-1,27
2006.442.50	8,19	8,22	0,0009	0,36
2006.442.51	6,67	6,60	0,0049	-1,06
2006.446.5	8,51	8,11	0,16	-4,93
			0,098425	TEM
			7,3874	Mean
			1,332337	%TEM
			0,009687	TEM^2
			0,599319	σ
			0,359183	σ ^2
			0,026971	TEM^2/ σ ^2
			0,973029	C.RELIABILITY (R)

TEM: technical error of measurement; %TEM: relative technical error of measurement; R: coefficient of reliability; σ: standard deviation
